# Prevalence of persons following a vegetarian diet in Germany

**DOI:** 10.17886/RKI-GBE-2016-039

**Published:** 2016-12-14

**Authors:** Gert B.M. Mensink, Clarissa Lage Barbosa, Anna-Kristin Brettschneider

**Affiliations:** Robert Koch Institute, Department for Epidemiology and Health Monitoring, Berlin, Germany

**Keywords:** NUTRITION, VEGETARIAN, HEALTH SURVEY, DEGS1, GERMANY

## Abstract

People adopt a vegetarian diet for various reasons. A largely plant-based diet not only has advantages for health, it also has positive social and environmental aspects. The aim of this analysis is to provide a description of the people in Germany who follow a predominantly vegetarian diet and to compare their food consumption with those of non-vegetarians. As part of DEGS1 (2008–2011), a validated questionnaire was used within a representative sample of 6,933 persons aged 18 to 79 to study how often and how much of 53 different food groups was consumed during a four-week period. The questionnaire also included a question about a vegetarian diet. The data were analysed descriptively and with a binary-logistical regression model. In Germany, 4.3% of the population (6.1% of women and 2.5% of men) aged 18 to 79 usually follows a vegetarian diet. The highest proportion of vegetarians is found among 18- to 29-year-olds (women 9.2% and men 5.0%) and among women aged 60 to 69 (7.3%). People with a higher level of education are more likely to usually follow a vegetarian diet. The same applies to people who live in large cities and those who conduct more than four hours of sports per week. In addition, women and men who usually follow a vegetarian diet not only consume significantly less meat compared with non-vegetarians, they also drink less energy-reduced drinks, and less beer and wine; they also drink more tea and eat more fruit and vegetables. A vegetarian lifestyle is often associated with positive socio-political impacts. It can, among others, contribute to a reduction in factory farming, which means it can help preserve the environment. A reduction in meat consumption in Germany would also be beneficial from a public health perspective, since meat consumption is currently considerably higher than the amounts recommended by the German Nutrition Society. The benefits linked to a vegetarian diet would be further strengthened, if, in addition to the relatively small group of people who completely refrain from eating meat, a larger section of the population would reduce their meat consumption.

## 1. Introduction

People decide to follow a vegetarian diet for various reasons. Common motives include ethical and moral concerns, which are also embedded in some religions. In this particular case, respect for every living being, a rejection of killing animals, and of causing suffering play significant roles. But vegetarianism can also be grounded on environmental reasons: a vegetarian diet can help reduce factory farming and lower methane and CO_2_ polution. Furthermore, a vegetarian diet also conserves the energy and water that would otherwise have been needed for animal farming. Vegetarianism can, therefore, promote a more compassionate manner of handling the environment [1], and a focus on plant-based products could even help provide the world’s population with sufficient food. Lastly, individual considerations about improving personal health can also play a role in the decision to adopt a vegetarian diet.

There are several types of vegetarian diet including ovo-lacto-vegetarianism, lacto-vegetarianism and veganism. In addition, there are types of diet which are based on largely plant-based foods, including large amounts of fruit and vegetables, but still include certain amounts of meat (flexitarianism) or fish (pesco-vegetarianism) (Table 1) [2, 3]. The reasons for vegetarianism described above play an important role in the decision about which type of diet an individual may choose to adopt. Industry has recognised the growing trend towards vegetarianism and has expanded its range of food products to include a wide variety of vegetarian and vegan products; these are becoming increasingly common in supermarkets and discount chains in Germany. Over the last few years, it has become far easier to eat a balanced vegetarian diet in Germany, and, today, vegetarians are more than just an idealistic minority in this country.

The vegetarian diet has a long history. The first written references to vegetarianism in Europe can be found in ancient Greece around 600 BC. The first German vegetarian association was founded in 1867 [4].The first German studies focusing on the impact of a vegetarian diet on health, particularly in relation to cancer and cardiovascular diseases, were conducted in Heidelberg, Gießen, and Berlin in the 1970s and 1980s [5–7].

### 1.1 The impact of the vegetarian diet on health

There was an initial assumption within the fields of nutrition and health science that vegetarians might have a higher risk of nutritional deficiencies [4]. In fact, it is more difficult to gain enough of certain nutrients from a vegetarian, or especially a vegan diet, than from a mixed diet. An adequate supply of vitamin B_12_ can be particularly problematic [8]. Vitamin B_12_ deficiency is associated with various neurological conditions and an increased risk of cardiovascular diseases [9]. The intake of the following nutrients can also be critical in a vegetarian diet: long-chain n-3 fatty acids, vitamin D, iron, calcium, zinc, iodine and selenium [3, 4, 10]. However, over the last few years, observations have concluded that vegetarians are not more likely to suffer from deficiencies of some of these nutrients than non-vegetarians [4, 11]. In particular, folate intake among vegetarians is usually higher than among non-vegetarians [4]. Recent studies have observed a generally healthy nutritional balance among people who follow a vegetarian diet and especially among those who follow a vegan diet. The Oxford-EPIC study showed that vegans have higher intakes of polyunsaturated fatty acids, unsaturated fatty acids and fibre. The overall better quality of fats ingested by vegans is due to their higher consumption of plant-based foods [12–14]. Nevertheless, vegans do need to ensure adequate levels of critical nutrients, especially vitamin B_12_, by taking dietary supplements or eating fortified foods [11, 15, 16].

The potential of a vegetarian, or mostly vegetarian, diet to reduce the risk of chronic diseases such as obesity, type 2 diabetes mellitus, cardiovascular diseases and cancer is currently emphasized [17]. A review based on several studies has shown that the prescription of a vegetarian diet can help to reduce the body mass index (BMI) [18]. A recent meta-analysis showed that a vegetarian diet is associated with a lower risk of ischemic heart diseases in both genders. It also identified a significant reduction in cerebrovascular diseases and all-cause mortality, but only among men [11]. Nevertheless, the health benefits associated with a vegetarian diet have mainly been shown in studies conducted on members of the Seventh-day Adventist Church [11, 17, 19, 20]. Most of these individuals follow, for religious reasons, a vegetarian diet and they also tend to live a generally healthier lifestyle than the overall population: they are less likely to smoke; they do not drink alcohol, and are more physically active. The impact of these lifestyle factors on the correlation between diet and health could not be completely disentangled until now.

The health benefits associated with a vegetarian diet are probably not only due to the high proportion of plant-based foods consumed by vegetarians and vegans; they are also due to the exclusion of animal products from vegetarian diets. A number of studies have demonstrated an independent association between a high level of consumption of animal products, especially processed red meat, and a higher risk of all-cause mortality [16, 19, 21]. In general, the German Nutrition Society (DGE) considers a vegetarian diet appropriate to adapt permanently. However, the DGE recommends that pregnant and breastfeeding women, as well as infants, children and adolescents, should avoid a vegan diet as it can be more difficult to obtain an adequate supply of some nutrients [22]. In contrast, the US Academy of Nutrition and Dietetics (AND; formerly known as American Dietetic Association) provides a different recommendation in this respect: the AND states that well-planned vegetarian and vegan diets are appropriate for individuals during all stages of life. Moreover, the AND also points out that a vegetarian diet can actually be beneficial because it helps prevent and treat certain diseases [23].

### 1.2 The prevalence of the vegetarian diet in Germany

In general, meat consumption in Germany has steadily reduced since 1990 [24]. Estimates of the prevalence of vegetarianism in Germany over the last 20 years have varied between 2% and 10%. The German National Health Interview and Examination Survey 1998 (GNHIES98) showed that about 8% of women and 3% of men exclusively or predominantly follow a vegetarian diet [25]. In 2006, the German National Nutrition Survey II (NVS II) indicated that approximately 2% of the population in Germany between 14 and 80 years of age was vegetarian [26]. An online survey conducted in 2013 showed that about 4% of the population is vegetarian; flexitarians were estimated at around 12% [27]. In contrast, the German Vegetarian Union (VEBU) estimates approximately 10% of the population as vegetarian, and 1% as vegan [28]. A European comparison of selected countries observed the highest numbers of vegetarians in Germany, Britain and Italy (9%), with relatively low numbers of vegetarians in France, Switzerland, and Austria (3%) [4].

The German Health Interview and Examination Survey for Children and Adolescents (KiGGS baseline survey, 2003–2006) showed that less than 2% of boys aged 3 or over and 3% of girls in the same age group consumed no meat, poultry or sausage [29, 30]. Among 14-to 17-year-olds this was as much as 2% of boys and 6% of girls. There are more young vegetarians in medium-sized and large cities. Children and adolescents with an immigration background are also more likely to be vegetarian [31].

The German Health Interview and Examination Survey for Adults (DEGS1, 2008–2011), which was conducted by the Robert Koch Institute, also collected information about vegetarian diets. The aim of the following analysis is to provide the prevalence of vegetarians in Germany and to describe the distribution of the vegetarian diet according to a number of selected characteristics. In addition, the food consumed by vegetarians is compared with that of non-vegetarians.

## 2. Methods

### 2.1 Dietary assessment in the German Health Interview and Examination Survey for Adults (DEGS1 )

DEGS1 was carried out between 2008 and 2011 and is part of the Robert Koch Institute’s health monitoring system. The concept and design of DEGS1 are described in detail elsewhere [12, 32, 33]. As part of DEGS1, comprehensive questionnaires, examinations, and tests were conducted among a representative sample of 18-to 79-year-old-population. Food consumption data was gathered using a semi-quantitative food frequency questionnaire (FFQ). This FFQ is a validated instrument that records the frequency and the portion size of a total of 53 food groups consumed over a four-week period [34]. The questionnaire was a further development of the FFQ used as part of the KiGGS baseline study [35]. In DEGS1, the questionnaire was completed by the study participants who also took part in the physical examination, and is available for a total of 7,115 people. It included the question: ‘How many times have you eaten (or drunk) …?’ for each of the 53 food items. Answers on how often a particular type of food was eaten could be provided as follows: ‘never’, ‘once a month’, ‘2–3 times per month’, ‘1–2 times per week’, ‘3–4 times per week’, ‘5–6 times per week’, ‘once per day’, ‘twice per day’, ‘3 times per day’, ‘4–5 times per day’, and ‘more times than 5 times per day’. Serving sizes could be reported for example as ‘½ portion (or less)’, ‘1 portion’, ‘2 portions’, ‘3 portions’ or ‘4 portions (or more)’. Depending on the type of food, there was also the option to select ‘¼ portion’. Additional portion descriptions in different measures were provided depending on the type of food, such as a glass, cup, bowl, plate, slice or piece. In order to provide a better estimate of portion size, most of the questions were illustrated with a picture. Data on how often and how much of a particular food was consumed were used to calculate the average food consumption. Food items were categorized into food groups for the following analysis.

### 2.2 Collecting information on vegetarian diet

As part of the DEGS1 FFQ, the participants were also asked: ‘Do you usually follow a vegetarian diet?’ This question could be answered with ‘Yes’ or ‘No’. Valid responses to this question are available for 6,933 people. In the following, the group of people who answered ‘Yes’ are referred to as vegetarian. Respondents who answered ‘No’ are considered non-vegetarian. However, the wording of the question influences the possible distribution of vegetarianism as it uses the word ‘usually’, the definition of vegetarian in DEGS1, thus may, also include people who occasionally eat meat or fish.

### 2.3 Construction of further variables

The information provided by participants on educational qualifications was used to create educational categories in line with the ‘Comparative Analysis of Social Mobility in Industrial Nations’ index (CASMIN). This index takes into account the differences between vocational training and more general educational paths [36]. Additionally, socio-economic status was determined using an index based on data collected on education, training, professional status, and net household income, which was weighted according to household needs. This index enabled the respondents to be categorised into low, medium or high socio-economic status [15].

Sport activity during the last three months was assessed by asking the following question: ‘How often do you do sport?’ The answers were categorised into ‘I don’t do any sport’, ‘less than 1 hour a week’, ‘regularly, 1 to 2 hours a week’, ‘regularly, 2 to 4 hours a week’ and ‘regularly, more than four hours a week’. These categories were reclassified for purposes of the current analysis as ‘> 4 hours a week’ and ‘≤ 4 hours a week’. Using the information on the participant’s residency and the number of inhabitants in their local area, place of residence was categorised as ‘rural (<5,000 inhabitants)’, ‘provincial (5,000 to < 20,000 inhabitants)’, ‘medium-sized city (20,000 to < 100,000 inhabitants)’ and ‘large city (≥ 100,000 inhabitants)’.

The analyses were carried out using a weighting factor to correct the deviations within the net sample from the actual German population statistics (as of 31 December 2010) regarding age, gender, region, nationality, place of residence, and education. The analyses were conducted using the complex survey procedures available in SAS 9.4, taking account of the weighting factors and the effect of the cluster design.

## 3. Results

### 3.1 Socio-demographic characteristics of vegetarians in Germany

**Fig. 1:**
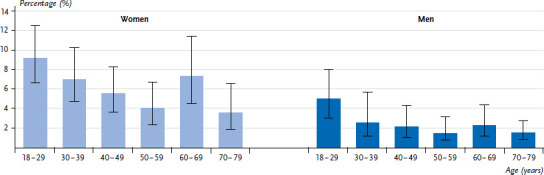
The proportion of 18- to 79-year-olds who usually follow a vegetarian diet according to gender and age Source: DEGS1 (2008–2011)

In Germany, 4.3% of adults aged 18 to 79 usually follow a vegetarian diet. A vegetarian diet is more common among women (6.1%) than men (2.5%) (Figure 1). The proportion of vegetarians is highest among 18- to 29-year-olds among both women (9.2%) and men (5.0%). The percentages reduce with increasing age, with the exception of women aged 60 to 69 since 7.3% of women in this age group usually follow a vegetarian diet.

Table 2 shows the percentages of women and men who usually follow a vegetarian diet according to socio-economic status, education, and community size. In women, a vegetarian diet is most common among those with a low socio-economic status; in contrast, men with a low socio-economic status are less likely to be vegetarian. A breakdown by education level, however, demonstrates that a higher proportion of both women and men with higher educational levels are more likely to usually follow a vegetarian diet. The proportion of people who usually follow a vegetarian diet is at its highest among both men and women who live in large cities.

According to the results of the binary logistic regression analyses, when gender, age, education, municipality size, and sporting activity is taken into account, women, people aged 18 to 29, people with a high education level, people who take part in sports, and people who live in large cities are significantly more likely to follow a vegetarian diet than the reference group (Table 3).

### 3.2 Food consumption by vegetarians

In the comparison of the average consumption of aggregated food groups in grams per day for women and men according to whether they usually follow a vegetarian or a non-vegetarian diet, it is observed that vegetarians not only eat significantly less meat than non-vegetarians, but they also consume less energy-reduced drinks, beer, and wine, and significantly more tea, fruit, and vegetables (Table 4). Women who usually follow a vegetarian diet, moreover, consume significantly less spirits, eggs, and pizza, and more dairy products compared with non-vegetarians. Men who usually follow a vegetarian diet consume significantly lower amounts of coffee and potatoes, and significantly more pasta and rice compared with non-vegetarians.

## 4. Discussion

Data from DEGS1 suggest that 4.3% of adults aged 18 to 79 in Germany usually follow a vegetarian diet. A vegetarian diet is significantly more common among women, young adults, people with a high education level, people who live in large cities, and among people who take part in sport for more than four hours per week.

The fact that many young women in particular are vegetarian has been observed in previous studies [25, 26]. Nevertheless, the relatively high proportion of vegetarian women among the 60- to 69-year-old age group is remarkable. A number of reasons could have a combined influence on this situation: one possible explanation could be that women in this age group no longer have to make as many compromises about their diet as they did when they had to give greater consideration to the preferences of other family members. In addition, their children may have convinced them to adopt a vegetarian diet. Health consciousness is also particularly high among this age group: an analysis of the data collected by the German Health Update (GEDA) shows that women over 60 are significantly more likely to care strongly or very strongly about their health than younger women [37]. Furthermore, they are more likely than men to adopt a vegetarian diet for health reasons following the diagnosis of a disease [38]. Finally, a cohort effect could also play a role to some extent: during the 1960s and 1970s, when these women were adolescents or young adults, Western Europe experienced a growing interest in the Far East and especially in Indian spirituality, meditation and, in connection also, vegetarianism [39].

The conclusion that a vegetarian diet is more common among individuals with a higher socio-economic status and people with a higher education level is also confirmed by other studies [40]. Within the DEGS1 results, it is remarkable that women with a low socio-economic status are significantly more likely to adopt a vegetarian diet than women with a mid-level socio-economic status. This can be explained by the fact that many young people have a low socio-economic status because they are still in training or at university and, thus, either have little or no income. This is also one of the reasons why the link to educational level was examined. A similar effect was not identified in this regard; instead, it is possible to draw a clear gradient with education, with a higher prevalence of vegetarianism among people with higher levels of education.

People who take part in sport more than four hours per week are more likely to follow a vegetarian diet than those who are less active. In addition, vegetarians were significantly more likely to state that they paid a lot of attention to achieve enough physical activity (results not shown). In fact, when vegetarians are compared with non-vegetarians, the statistically significant differences between the food choices those groups make, demonstrate a generally more health-conscious form of food consumption among vegetarians. These findings indicate that vegetarians live an overall healthier lifestyle than non-vegetarians. This has been observed in previous studies, including those conducted among the members of the Seventh-day Adventist Church mentioned above. However, irrespective of religion-based lifestyles, people who follow a vegetarian diet are generally less likely to smoke; they also drink less alcohol, and are more physically active [11].

However, the analysis of the vegetarian diet using data from DEGS1 has a number of limitations. Due to the low prevalence of vegetarianism and the complex study design, it is pointless to use other determinants to differentiate between the various forms of vegetarianism. The descriptive results largely demonstrate overlapping confidence intervals (Figure 1 and Table 2). Statistically significant associations become apparent in the multivariate analysis. Moreover, it is hardly possible to evaluate the additional information which could be used to differentiate between the various forms of vegetarianism. DEGS1 asked the supplementary question: ‘Which of the following foods do you not eat?’, with the option to select ‘meat, poultry, and sausage’, ‘fish’, ‘milk and dairy products’, and ‘eggs’. However, approximately half of the respondents who reported to follow usually a vegetarian diet skipped this question, and the subgroups are too small for specific evaluation.

Compared with other studies on vegetarianism, DEGS1 observed a slightly lower proportion of vegetarians among the population than suggested by the results of the German National Health Interview and Examination Survey 1998 (GNHIES98). This could be due to the different ways in which the studies formulated their questions about vegetarianism. GNHIES98 classified people who had been vegetarian in the past as currently vegetarian. In contrast, DEGS1 only took a participant’s diet at the time of the study into account. The fluctuating proportion of vegetarians and vegans among the German population could also be connected to the fact that meat scandals may have caused people to give up meat temporarily; this may have occurred in 2000, when beef consumption collapsed after the first German case of BSE was reported.

In recent years, other studies have identified a similar proportion of vegetarians and vegans in Germany as DEGS1 [25, 27]. The proportion of vegetarians and vegans has probably increased further in recent years. Nevertheless, the results of these studies varied considerably between 2% and 10% (see introduction) [26, 28]. This is partly due to the various definitions used by the studies and the way in which the vegetarian diet is measured. Some studies use reported food intake to define participants as vegetarian [41]. This procedure is particularly problematic because of the increasingly large number of vegetarian and vegan meat substitutes available. Thus, if a participant answered ‘Yes’ to a question about whether they eat sausages, they could be referring to vegetarian sausages; on the other hand, a simple question such as ‘Are you vegetarian?’ would not do justice to the variety of vegetarian diets. Therefore, it is probably more meaningful to ask direct questions about participants’ diet in future studies to enable an analysis of the different forms of a plant-based diet. Moreover, DEGS1 used the term ‘usually’ in the question about vegetarian diet. This widens the range of people it classifies as vegetarian, and may reduce the comparability with other study results. In contrast, the National Consumption Study II (NVS) estimated that 2% of the population are vegetarian, which is a significantly lower prevalence, but this study also operated with a much stricter definition of the term. Moreover, the NVS II also gathered detailed information about the type of vegetarian diet its participants were following (vegan, lacto-vegetarian, ovo-vegetarian, ovo-lacto-vegetarian, and ovo-lacto-vegetarian with fish, as well as a raw fruit and vegetable diet). No questions, however, were asked about a flexitarian diet. In contrast, the definition used in DEGS1 does take into account the fact that there are now many ‘flexitarians’ in Germany, and this could also explain the higher prevalence of the vegetarian diet in DEGS1.

**Infobox**

The German Nutrition Society (DGE) recommends a diversified diet which properly combines nutrient-rich and low-energy foods. Many of its guidelines can actually be achieved more easily through a plant-based diet; i.e.:

► At least 30 grams of dietary fibre daily.
► 5 servings of fruits and vegetables a day, preferably fresh.
► No more than 300–600 g of meat and meat products per week.

See: 10 guidelines for a wholesome diet by the DGE:

https://www.dge.de/index.php?id=322

Currently, only a relatively small number of people in Germany entirely omit the consumption of meat and fish. A greater contribution to achieving widely shared socio-political aims, such as protecting the environment or reducing levels of factory farming, could be achieved if a larger section of the population were to gradually reduce its consumption of animal products without necessarily giving up entirely the consumption of animal products. This trend would be desirable from a public health perspective, since average meat consumption in Germany is considerably higher than the level recommended by the DGE (see infobox). Moreover, a shift towards a vegetarian diet is expected to provide benefits to the health of the population [25, 42, 43].

## Key statements

- In Germany, 4.3% of the adults usually follow a vegetarian diet.
- Vegetarians consume significantly lower amounts of energy-reduced drinks, beer and wine, and significantly higher amounts of tea, fruit and vegetables than non-vegetarians.
- A vegetarian diet is more prevalent among women (6.1%) than men (2.5%).
- A vegetarian diet is most common among 18- to 29-year-old women and men, and women aged 60 to 69.
- The proportion of people following usually a vegetarian diet rises with increasing levels of education.

## Figures and Tables

**Table 1:** The various forms of the vegetarian diet

Name	Does not eat…
Ovo-lacto-vegetarian	…meat and fish products; this is what is usually meant by the term ‘vegetarian’
Lacto-vegetarian	…meat and fish products, eggs
Ovo-vegetarian	…meat and fish products, milk and dairy products
Pesco-vegetarian	…meat products
Flexitarian (normally vegetarian)	…meat and fish products; occasionally, however, small amounts of meat and fish products are consumed
Vegan	…all animal products (meat, fish, milk, eggs, honey)
Raw food	…all animal products (meat, fish, milk, eggs, honey) and cooked or processed food
Fruitarians/frugivores	…all animal products (meat, fish, milk, eggs, honey) and cooked or processed foods, including vegetables; only eats fruit, nuts and seeds

**Table 2:** The proportion of 18- to 79-year-olds who usually follow a vegetarian diet according to gender, socio-economic status, level of education and size of place of residence Source: DEGS1 (2008–2011)

	Women	Men
	**% (95% CI)**	**% (95% CI)**
**Socio-economic status**	**(n=3,594)**	**(n=3,300)**
Low	8.1 (5.5 – 11.7)	1.9 (0.7 – 4.7)
Medium	4.9 (3.8 – 6.3)	2.6 (1.7 – 3.7)
High	7.8 (5.3 – 11.4)	2.5 (1.6 – 3.9)
**Education**	**(n=3,652)**	**(n=3,359)**
Low	5.3 (3.8 – 7.3)	1.5 (0.8 – 2.9)
Medium	6.0 (4.6 – 7.7)	2.5 (1.6 – 3.7)
High	8.8 (6.1 – 12.7)	4.2 (2.6 – 6.8)
**Community size**	**(n=3,673)**	**(n=3,378)**
Rural (< 5,000)	4.6 (3.0 – 7.1)	1.3 (0.6 – 2.5)
Provincial (5,000 – < 20,000)	6.9 (4.7 – 10.2)	2.2 (1.1 – 4.4)
Medium-sized city (20,000 – < 100,000)	4.7 (3.3 – 6.8)	1.9 (1.1 – 3.3)
Large city (≥ 100,000)	7.4 (5.4 – 10.1)	4.0 (2.6 – 6.1)

CI = confidence interval

**Table 3:** Multivariate associations between a vegetarian diet and selected determinants for 18- to 79-year-olds (n = 6,745) Source: DEGS1 (2008–2011)

	OR (95% CI)
**Gender**	
Women	**2.9 (2.0 – 4.1)**
Men	Ref.
**Age**	
18 – 29 years	**2.7 (1.3 – 5.4)**
30 – 39 years	1.5 (0.8 – 2.9)
40 – 49 years	1.3 (0.6 – 2.6)
50 – 59 years	**0.9 (0.4 – 1.8)**
60 – 69 years	1.4 (0.7 – 3.0)
70 – 79 years	Ref.
**Education**	
Low	Ref.
Medium	1.1 (0.7 – 1.7)
High	**1.7 (1.0 – 2.9)**
**Community size**	
Rural (< 5,000)	Ref.
Provincial (5,000 – < 20,000)	1.1 (0.7 – 1.9)
Midium-sized city (20,000 – < 100,000)	1.0 (0.6 – 1.7)
Large city (≥ 100,000)	**1.6 (1.0 – 2.6)**
**Sport activity**	
> 4 hours/week	**1.7 (1.0 – 2.6)**
≤ 4 hours/week	Ref.

OR = odds ratio; CI = confidence interval; Ref. = Reference group Bold: significant (p < 0.05)

**Table 4:** Food consumption for 18- to 79-year-olds according to gender and whether they usually follow a vegetarian or non-vegetarian lifestyle Source: DEGS1 (2008–2011)

	Women (g/day)				Men (g/day)			
	vegetarians (n=190)		non-vegetarians (n=3,483)		vegetarians (n=79)		non-vegetarians (n=3,299)	
Food groups	M	% (95% CI)	M	95% CI	M	95% CI	M	95% CI
Milk	239	(187 – 291)	265	(246 – 285)	219	(108 – 331)	276	(253 – 299)
Fizzy drinks and fruit juice	330	(198 – 462)	377	(336 – 417)	397	(106 – 689)	561	(514 – 609)
Energy-reduced drinks	**93**	(1 – 185)	111	(87 – 134)	**46**	(16 – 76)	118	(91 – 144)
Vegetable juice	14	(4 – 25)	7	(6 – 8)	14	(-5 – 34)	6	(5 – 7)
Water	1,826	(1,484 – 2,168)	1,779	(1,700 – 1,859)	1,644	(1,186 – 2,102)	1,386	(1,319 – 1,452)
Tea	**590**	(439 – 740)	364	(332 – 395)	**530**	(270 – 791)	209	(186 – 232)
Coffee	430	(320 – 540)	478	(451 – 505)	**358**	(249 – 468)	501	(473 – 529)
Beer	**23**	(13 – 34)	38	(33 – 44)	**63**	(30 – 96)	256	(225 – 286)
Wine	**20**	(14 – 26)	30	(28 – 33)	**24**	(14 – 33)	34	(29 – 38)
Spirits	**3**	(1 – 5)	9	(9 – 12)	12	(3 – 21)	13	(11 – 15)
Cereals	6	(4 – 8)	5	(4 – 5)	12	(6 – 18)	6	(5 – 7)
Bread	136	(118 – 154)	137	(132 – 142)	169	(126 – 212)	183	(176 – 191)
Spreadable fats	7	(6 – 9)	9	(8 – 9)	10	(7 – 12)	11	(11 – 12)
Dairy products	**155**	(131 – 180)	123	(118 – 128)	159	(35 – 283)	112	(106 – 118)
Sweet spreads	11	(9 – 13)	10	(10 – 11)	25	(-5 – 56)	11	(10 – 12)
Eggs	**10**	(8 – 12)	14	(14 – 15)	32	(10 – 55)	19	(18 – 21)
Meat and sausages	**27**	(18 – 37)	88	(85 – 91)	**70**	(30 – 110)	138	(132 – 144)
Fish	13	(8 – 18)	17	(16 – 18)	30	(12 – 48)	19	(17 – 20)
Fruit	**451**	(349 – 554)	250	(236 – 265)	**267**	(189 – 344)	182	(173 – 191)
Vegetables	**206**	(177 – 235)	154	(147 – 160)	**174**	(140 – 208)	111	(106 – 116)
Pasta and rice	47	(41 – 54)	45	(44 – 47)	**83**	(60 – 106)	52	(49 – 55)
Potatoes	88	(67 – 108)	90	(86 – 94)	**79**	(58 – 101)	104	(98 – 110)
Pizza	**12**	(9 – 14)	15	(14 – 16)	38	(22 – 53)	24	(23 – 26)
Cake	24	(19 – 29)	28	(26 – 30)	40	(27 – 54)	33	(31 – 35)
Confectionery	33	(27 – 40)	38	(35 – 40)	35	(22 – 48)	40	(36 – 44)
Savoury snacks	2	(1 – 3)	4	(2 – 6)	6	(1 – 10)	4	(4 – 5)
Nuts	3	(2 – 4)	2	(2 – 2)	6	(1 – 12)	2	(2 – 2)

CI = confidence interval; M = mean

Bold: significant (p < 0.05)
